# Why we do what we do. A brief analysis of cancer therapies

**DOI:** 10.17179/excli2020-2972

**Published:** 2020-10-28

**Authors:** Carlos M. Galmarini

**Affiliations:** 1 Topazium Artificial Intelligence. Paseo de la Castellana 40 Pl. 8, 28046. Madrid, Spain

**Keywords:** neoplasm, molecular targeted therapies, immunotherapy, artificial intelligence, antiangiogenic agents

## Abstract

The goal of all medical activity is to preserve health in fit people, and to restore the sick into a state of complete physical, mental and social wellbeing. In an effort to determine whether we are achieving this last goal in oncology, herein we review the biological and clinical framework that has led to the foundations of the current anticancer treatment paradigm. Currently, cancer therapy is still based on the ancient axiom that states that the complete eradication of the tumor burden is the only way to achieve a cure. This strategy has led to a substantial improvement in survival rates as cancer mortality rates have dropped in an unprecedented way. Despite this progress, more than 9 million people still die from cancer every year, indicating that the current treatment strategy is not leading to a cancer cure, but to a cancer remission, that is “the temporary absence of manifestations of a particular disease”; after months or years of remission, in most patients, cancer will inevitably recur. Our critical analysis indicates that it is time to discuss about the new key challenges and future directions in clinical oncology. We need to generate novel treatment strategies more suited to the current clinical reality.

## Introduction

If we ask medical students or physicians around the world why they study medicine, the answer would be almost unanimous: “to cure those who are sick and save their lives”. But what does it really mean to cure the sick? According to the dictionary, the verb “to cure” is defined as “to restore to health” (Thesaurus, 2019[63]). However, what is health? The World Health Organization (WHO) defines health as “*a state of complete physical, mental and social wellbeing and not merely the absence of disease or infirmity*” (WHO, 2019[69]). We can then redefine the action of curing somebody as “to restore to a state of complete physical, mental and social wellbeing”. In order to achieve this goal, throughout history, the medical community has developed many different therapeutic strategies that were based on the scientific paradigms available at the time. Scientific paradigms are produced by clustering information and knowledge generated over time. Indeed, a scientific paradigm is the framework containing the basic assumptions, approaches, and methodologies that are commonly accepted by the members of a scientific community. The aim of a scientific paradigm is to provide concrete solutions to specific problems. A scientific paradigm is a model to understand reality, but it is not the reality. When scientists claim that unresolved problems are often untreatable, they begin to question the prevailing paradigm and seek to replace it (Kuhn, 1970[36]). In an effort to determine whether the current cancer paradigm is achieving the goal of restoring cancer patients to a state of complete physical, mental and social wellbeing, herein we review the biological and clinical framework that has led to the foundations of the current cancer treatment and prevention strategies. Our critical analysis of the medical results obtained with the current paradigm indicates that it is time to replace it to generate novel treatment strategies more suited to the current clinical reality. 

## The Current Cancer Treatment Paradigm: “For Extreme Diseases, Extreme Methods of Cure”

“In treating dangerous, acute diseases, when life's flame flickers at the gates of death, do not hesitate to use heroic measures: they may avail and save your patient's breath” (Hippocrates, Aphorisms, Section 1 A6) (Scholtz, 1940[56]).

The current cancer treatment paradigm is based on the Hippocratic view that supports that to cure severe diseases, extreme therapeutic approaches are the best. In cancer treatment, this paradigm has evolved along history, but always with the same ultimate goal: to cure cancer through the complete eradication of the tumor burden. In order to achieve this goal, certain combinations of surgery, radiotherapy, systemic chemotherapy, and biological and hormonal therapies are used to control the local and systemic components of the disease. Three central axioms that will be explained in detail support this paradigm (Table 1(Tab. 1)).

### Axiom #1: Cancer is an anatomical condition: “a chance to cut is a chance to cure” (Matmos, 2001)

The initial approach to treat cancer was purely anatomical, i.e., it was based on the eradication of the tumor mass by means of its resection or cauterization. It was not until the late 19^th^ century, when great strides were made in general surgery (anesthesia, antisepsis and blood transfusions), that the anatomical approach became the first potentially curative anticancer treatment (Gawande, 2012[26]). Great masters such as Theodor Billroth (1829-1894), William Halsted (1852-1922), Harvey Cushing (1869-1939), Ernst Wertheim (1864-1920) or Allen Whipple (1881-1963), amongst others, designed and developed surgical techniques for each specific cancer type in order to remove the entire tumor along with the lymph nodes around the tumor. Nowadays, many of these surgical procedures continue to be in use. Radiotherapy can be compared to an invisible blade and therefore, it can be considered as another anatomical approach to cure cancer. Since 1896, when German physicist Wilhelm Roentgen (1845-1923) discovered X rays, different types of radiation have been used to treat cancer (Gianfaldoni et al., 2017[27]). Later, in 1898, Marie (1867-1934) and Pierre Curie (1859-1906) isolated radium, an element that had the ability of depositing radiation deep inside tissues (Curie et al., 1898[11]). This discovery led to a golden period of radiotherapy to treat patients affected by deep cancers. 

However, during the last decades of the 20^th^ century, and despite remarkable scientific progress, only a small fraction of patients with locally restricted cancers (e.g., primary tumors without metastatic lesions) could be cured with these therapeutic modalities. Most tumors returned after surgery or radiotherapy, even if more aggressive operations were performed or higher doses of radiation were applied. This was not surprising. Ancient physicians and surgeons knew that cancer patients usually relapsed after the tumor was surgically resected. Hippocrates wisely understood that “*what remains in diseases after the crisis is apt to produce relapses*” (Hippocrates, Aphorisms, Section 2 A12) (Scholtz, 1940[57]). Moreover, interventions could be more harmful than no treatment at all. The Roman surgeon Aulus Cornelius Celsus (25 BC - 50 AD) in his general encyclopedia De Artibus wrote: “*We reject any treatment of the latter stages, be it by caustic methods, cauterization or the scalpel. Any aggressive measure would only irritate the process and, even if the surgeon succeeded in healing the operation, the disease would inevitably recur; successful treatment would only be possible in the first stage*” (Celsus, 30; Kockerling et al., 2013[34]). It is now clear that cancer is not an anatomical condition and therefore, anatomical therapeutic strategies such as surgery or radiotherapy can only cure a small proportion of cancer patients; for the vast majority, these treatments are only palliative. Based on this, the role of surgery was redefined and currently, surgeons have developed greater technical expertise in minimizing the amounts of healthy tissue being removed during cancer surgery. Similarly, nowadays, radiation can be aimed more precisely. Small tumors in early stage cancers can be resected without extensive amputation of healthy tissues, while late stage tumors cannot be eradicated with these procedures and thus, it makes no sense for patients to undergo aggressive surgeries as a palliative treatment. In summary, eradicating tumors by removing tissues or organs has not translated into a cure for most cancer patients.

### Axiom #2: Cancer is a genetic disease: “The myth of Achilles”

The seeds for establishing a relationship between genetics and cancer were planted in the beginning of the 20^th^ century by imaginative scientists such as Theodor Boveri (1862-1915) (Boveri, 2008[6]). Boveri was ahead of his time and used experimental evidence to develop the concepts that underpin much of what is currently considered the basic tenets of cancer genetics. Boveri's chromosome theory of genetic inheritance served as the starting point for his tumorigenesis model and the description of the key hallmarks of cancer, such as chromosomal instability, tumor heterogeneity, faulty tumor suppressor genes and tumor clonality (Hansford and Huntsman, 2014[28]; Ried, 2009[51]). The bulk of the evidence generated in the following decades confirmed Boveri's ideas. Nowadays, it is believed that cancer is a genetic disease, the result of somatic evolution, wherein a single clonal lineage acquires driver mutations that enables cells to circumvent constraints on cell proliferation, and finally becoming cancerous (Bishop, 1987[4]; Nowell, 1976[47]; Weinberg, 1989[67]). A minimum of three to seven of such mutations appear to be required to complete this process (Miller, 1980[45]). Since the establishment of the first model of colorectal cancer by Bert Vogelstein (1949- ), there was an abundance of research identifying and validating key genes and pathways that are dysregulated or mutated in certain tumor types (Lengauer et al., 1998[38]; Vogelstein and Kinzler, 1993[65]). More recently, it has been accepted that the genetic variability across cancer cells is also affected by epigenetic changes, such as DNA methylation. Tumors are now considered as complex systems composed of multiple cell subclones, with increasing layers of genomic complexity and heterogeneity.

The advantages conferred by the acquisition of driver mutations leading to tumorigenesis also exposed “vulnerabilities” in cancer cells that are absent in healthy cells. It was then hypothesized that these vulnerabilities could be considered as the “Achilles heel” of cancer cells and exploited to target them whilst sparing healthy cells. This postulate was based on the old concept of the "poisoned arrow" developed by Paul Ehrlich (1854-1915) (Ehrlich, 1913[15]). Indeed, Ehrlich assumed that destructive toxins developed their injurious action on parasites by binding to certain specific components that he named "chemoreceptors”. Thus, he compared the ideal chemotherapeutic agents to a “poisoned arrow”: the fixing group of the drug, which anchored itself to the chemoreceptor of the parasite, corresponded to the arrowhead, and the warhead group was the poison smeared on the arrowhead. According to Ehrlich, the best “poisoned arrow” should include a chemical group specific to the chemoreceptors of the parasites, with no analogue in the organs of the body, thus delivering the poison only to the parasite. This would allow what Ehrlich named as the “therapia sterilisans magna”, which consisted in freeing the organism from the parasites without affecting body tissues.

Although originally developed for antibiotics, the “poisoned arrow” strategy was later applied in cancer chemotherapy. This began with the clinical use of folic acid antagonists and nitrogen mustards in the middle 40s of the 20^th^ century (for review, see Galmarini et al., 2012[25]). These molecules primarily targeted biochemical pathways involved in cell proliferation. With the discovery of different oncogenes, tumor suppressor genes and signaling pathways that were involved in carcinogenesis (“cancer chemoreceptors”), classical chemotherapy transitioned to what is known as “targeted therapies”. Targeted therapies consist in tailor-made molecules that inhibit or modulate the very genetic mechanism underlying the neoplasm, enabling a selective cancer treatment with minimal side effects. It was believed that this “poisoned arrow” (mutated into “magic bullet”) strategy would lead to a cancer cure. Several targeted therapies were developed showing unparalleled activity and became the standard of care for patients having tumors with matching molecular profiles (Dancey et al., 2012[12]). These novel selective therapies changed completely the cancer treatment landscape. Traditionally, tumors from the same anatomical site were treated as one tumor type. However, in the last decades, this notion has been replaced by the concept of targeting driver pathways in tumors from different anatomical sites. This is studied in basket or umbrella trials, which are designed to test the effect of one drug on a certain molecular alteration in a variety of tumor types (Redig and Janne, 2015[50]; Stenzinger et al., 2015[61]). 

**Figure 1:**
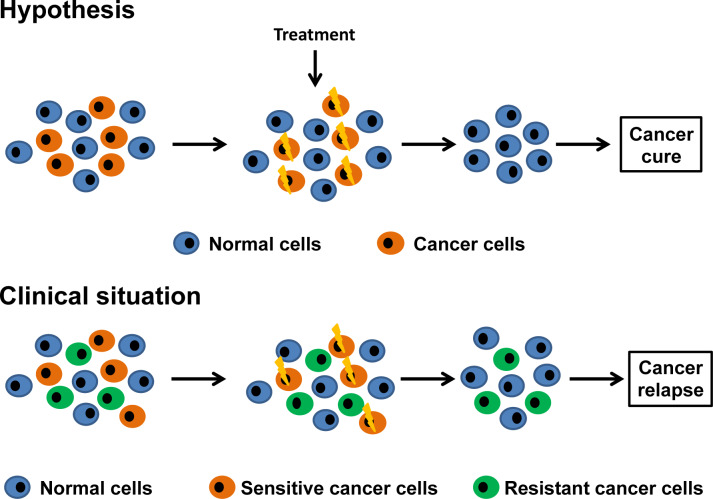
The “magic bullet” strategy. Although based on the concept that cancer cells present “vulnerabilities” that are absent in healthy cells and thus, can be exploited as therapeutic targets, the “magic bullet” strategy failed to achieve the goal of curing most cancer patients. Indeed, tumors can be resistant to “targeted” therapies at presentation or become resistant after an initial response.

However, despite advances in the molecular design of chemotherapeutic agents, most cancers are resistant to therapy at presentation or become resistant after an initial response (Figure 1(Fig. 1)). Indeed, “targeted” therapies are facing the same drug resistance problems as conventional chemotherapeutic agents. When the problem is analyzed in depth, it appears that the causes for the failure in chemotherapy are multifactorial, with many factors influencing response to treatment (Galmarini et al., 2012[25]; Tredan et al., 2007[64]). To overcome this problem, second- and third-generation selective agents have been developed for clinical use. However, unfortunately, even when treated with these novel inhibitors, tumors become resistant. Thus, drug resistance remains a problem that limits the clinical use of “classic” and “targeted” drugs. Although also based on the concept that cancer is a genetic disease, the “magic bullet” strategy targeting the “Achilles heel” of cancer cells equally failed to achieve the goal of curing most cancer patients.

### Axiom #3: Cancer is a microenvironmental disease: “The forest, not the tree”

The efficacy of drugs targeting distinct cancer driver pathways varies significantly across cancer patients, and this could not only be ascribed to cancer genetics. This is not surprising, as tumors are not just a cluster of mutated cells, but organ-like structures with many different components, such as non-malignant lymphoid and/or myeloid cells, as well as fibroblasts, endothelial cells, pericytes, and blood and lymphatic vessels, all embedded within the tumor stroma. All these components are interlinked by a vast array of cytokines, chemokines and growth factors that constitute the tumor microenvironment (TME) (Schaefer and Serrano, 2016[54]). This concept is not new. More than a century ago, Stephen Paget (1855-1926) proposed a “seed and soil” hypothesis suggesting that the tendency of tumor metastases to develop in specific organs was due to favorable interactions between cancer cells (the “seed”) and the organ microenvironment (the “soil”) (Paget, 1889[48]). An extensive body of clinical data and experimental research has confirmed Paget's “seed and soil” hypothesis (Fidler and Poste, 2008[18]). Indeed, the evolution of neoplasms is determined not only by the genetic features of cancer cells, but also by the selective pressure of their TME, which determines what changes provide adaptive benefits to the tumor (Maley et al., 2017[40]). The advantages conferred by the TME heterogeneity driving tissue-specific tumorigenesis also exposed vulnerabilities in tumors that may be considered as an “Achilles heel” and may be exploited as specific treatment targets, whilst sparing healthy tissues (Schneider et al., 2017[55]). The two tumor susceptibilities that could be targeted based on TME heterogeneity were tumor angiogenesis and the immune system.

#### Attacking tumor angiogenesis: tumors under siege

Until the 70s, it was believed that tumor growth was primarily supported by actively recruiting blood vessels from the surrounding tissue. On the other hand, some publications claimed that the growth of solid tumors was always accompanied by neovascularization (Algire and Chalkley, 1945[2]; Feigin et al., 1958[17]). In this context, Judah Folkman (1933-2008) hypothesized that most solid tumors initially exist as a small cluster of cells that eventually expands to a size of approximately 1-3 mm^3^. At this stage the cells enter into a dormant state, as simple diffusion of nutrients is no longer sufficient for tumors of this size (Folkman, 1971[19]). Cancer cells then acquire the ability to release an angiogenic mediator (tumor angiogenesis factor or TAF) that stimulates the rapid formation of new capillaries around the tumor (Folkman et al., 1971[21]). Only then can the tumor continue to grow. Folkman thus proposed to treat cancer through an “anti-angiogenic” approach: blockade of TAF activity to inhibit the formation of new blood vessels around the tumor, inducing a permanent non-vascularized dormant state. Thus, Folkman's group started to avidly research for angiogenic inhibitors. The first inhibitor was found in a cartilage and suppressed tumor growth when it was infused into the vascular bed of murine and rabbit tumors (Brem and Folkman, 1975[7]; Langer et al., 1980[37]). Many other angiogenic inhibitors were subsequently discovered (Folkman and Ingber, 1992[20]). Finally, the first clinical success in anti-angiogenic therapy came with the use of α-interferon as a treatment for hemangioma in infants and newborns (Ezekowitz et al., 1992[16]; White et al., 1989[68]). 

During that period, other research groups started looking for new angiogenic factors, and a dozen of them were identified in tumor and healthy tissues. The most famous angiogenic factor was discovered in 1983 by Harold Dvorak (1937- ) et al. when they isolated what they named as the “vascular permeability factor” (VPF) (Senger et al., 1983[59]). Lately, in 1989, Napoleone Ferrara (1956- ) et al. sequenced and characterized what they named as the “vascular endothelial growth factor” (VEGF), which turned out to be VPF (Leung et al., 1989[39]). VEGF/VPF is an endothelial cell mitogen regulated by hypoxia. Later on, Ferrara et al. identified the high-affinity tyrosine kinase receptor for VEGF (de Vries et al., 1992[13]). Ferrara's group additionally demonstrated that anti-VEGF monoclonal antibodies neutralized human VEGF and, when injected subcutaneously to nude mice, exerted a potent inhibitory effect on cancer cell growth in several tumor cell lines (Borgstrom et al., 1998[5]). In 2004, the first anti-angiogenic therapy, bevacizumab, was approved for cancer treatment. Nowadays, several antiangiogenic drugs targeting VEGF or other components of the angiogenic pathway are used to treat cancer (Cao et al., 2011[8]).

**Figure 2:**
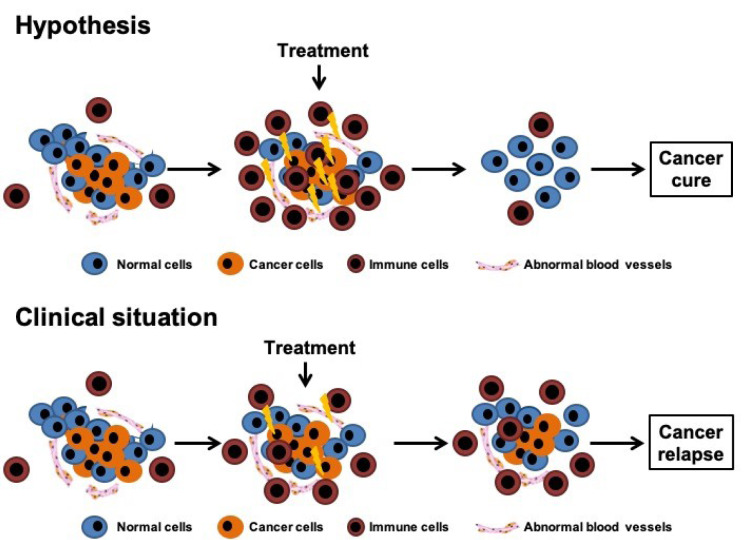
Microenvironmental strategies. Tumors are organ-like structures and thus targeting the tumor microenvironment can show profound effects in cancer treatment. However, current antiangiogenic and modern immunotherapies produce modest benefits as a high proportion of patients who initially respond subsequently relapse.

However, unlike the results obtained in most preclinical tumor models, current antiangiogenic therapies produce only modest benefits when administered as a monotherapy; clinical benefits with antiangiogenic therapies are usually achieved by combining them with existing chemotherapy (Kamrava et al., 2009[33]; Plum et al., 2003[49]). Moreover, a proportion of patients who initially respond to an antiangiogenic therapy subsequently relapse (Figure 2(Fig. 2)). These clinical findings demonstrate that tumors present a high degree of intrinsic resistance to antiangiogenic therapies or that they can acquire this resistance following drug treatment, as observed with chemotherapy. The causes of antiangiogenic resistance are not yet understood, but it is likely to arise from compensation by other angiogenic factors and redundancy in angiogenic stimulators, which may allow to overcome the blockade of a particular component in the angiogenic pathway (Bergers and Hanahan, 2008[3]).

#### Fostering the immune system: calling for reinforcements

The ability to circumvent local and systemic immune surveillance mechanisms is an essential step in tumor evolution. The TME plays an important role in the circumvention of the immune system by cancer cells. This is so because TMEs consist of an immune infiltrate that is dominated by immunosuppressive cell types, such as regulatory T cells (Tregs), M2-phenotype macrophages and myeloid-derived suppressor cells (MDSCs) (Fridman et al., 2017[22]). Cancer cells take advantage of these particular characteristics of TMEs to avoid, subvert and circumvent the immune system (Slaney et al., 2013[60]). Thus, restoring antitumor immunity seems to be a plausible strategy for cancer treatment.

In the late 19^th^ century, William Coley (1862-1936) noticed that in a considerable number of patients with unresectable cancers who also suffered from accidental erysipelas (usually by *Streptococcus pyogenes*), tumors rapidly decreased in size or even disappeared, with some patients remaining well for many years (Coley, 1910[10]). Based on these clinical observations, Coley decided to administer live cultures of streptococcus of erysipelas for the treatment of sarcomas. His first inoculation was performed in a patient suffering from an unresectable, recurrent spindle-cell sarcoma of the right tonsil; the patient also had a large metastatic tumor in the right cervical region. Coley injected 5 decigrams of a bouillon culture of streptococcus of erysipelas obtained from Koch's laboratory in Germany. The patient developed a severe erysipelas crisis, nearly causing his death. However, after two weeks, the neck tumor incredibly disappeared, and the tonsil tumor decreased in size. Coley then treated this way ten additional patients with unresectable and advanced sarcomas and carcinomas. Again, in all cases, he observed a significant decrease in tumor size (Coley, 1910[10]). Coley then realized that in order to achieve a therapeutic effect, it was not necessary to generate an erysipelas crisis, as the therapeutic activity of the erysipelas was due to toxic bacterial products, not to the bacteria itself. These toxins produced certain changes in the blood or serum (e.g., fever and subsequent leukocytosis) that restored the weakened or lost immunity (Coley, 1928[9]). In total, Coley treated more than 500 patients. Nearly all of them exhibited a clinical improvement, but the effect of treatment gradually declined until no longer being effective, with a fatal outcome of the disease (Coley, 1928[9]). In any case, Coley advocated the use of bacterial toxins for the treatment of all cases of unresectable sarcoma, and after all surgeries for primary sarcomas or carcinomas, as a prophylactic measure against recurrence. The obstacles for further developing Coley's treatment were primarily the difficulty in obtaining a toxin preparation with a uniform standard and the occurrence of several cases with a fatal outcome when other physicians applied this treatment. Due to this, the use of Coley's toxin approach gradually disappeared from the clinical setting.

Many years later, Georges Mathé (1922-2010) resurfaced Coley's ideas (Watts, 2010[66]). As other physicians, Mathé believed that a cancer true remission would only be achieved after the total eradication of all cancer cells. Although surgery, radiotherapy and chemotherapy induced a substantial reduction of the total tumor cell mass, there were always residual living cancer cells that remained and, after a certain period of time, caused the recurrence of the disease. The challenge lied in achieving a state of complete remission by minimizing the number of residual cancer cells without prolonging the duration of treatment, as this would increase the risk of developing a resistant cell subpopulation, which would eventually lead to a relapse. Mathé thought that the only way to avoid tumor recurrence after conventional treatments was to eliminate up to the last cancer cell (Mathé, 1974[42]). This may only be achieved by increasing the body's ability to detect and destroy residual cancer cells by therapeutic stimulation of the immune system, a concept that he named “active immunotherapy”. Mathé recovered Coley's strategy of using live bacteria to treat malignancies but, unlike Coley, Mathé had access to worldwide tested and industrially manufactured attenuated vaccines. In the early 60s, Mathé began to work with the Bacillus Calmette-Guérin vaccine (BCG) to achieve the unspecific activation of the immune system as a way to further treat cancer after chemotherapy (Mathé, 1968[41]). This approach showed mixed success for different tumor types and nowadays, intravesical BCG infusions are still a major component of standard treatment in bladder cancer (Kamat et al., 2016[32]; Mathé et al., 1973[43]). 

From 1980 and during the course of the following three decades, several immunological approaches were tested for the treatment of leukemia and solid tumors. In the late 80s, metastatic cancer patients have started to be treated with large doses of interferons and IL2 to enhance T-cell production. A decade later, patients with non-Hodgkin's lymphomas received the first monoclonal antibody, rituximab, as treatment. Currently, immunotherapy has become one of the pillars of cancer treatment, providing the unprecedented opportunity to, in some cases, cure several types of malignancies (Fridman, et al., 2017[22]). Most of these groundbreaking immunotherapies consist of monoclonal antibodies that block T-cell checkpoint receptors and their cognate ligands (e.g., ipilimumab, pembrolizumab, nivolumab) (Adams et al., 2015[1]). Recently, genetically engineered autologous dendritic cell therapies (sipuleucel-T) or T-cell therapies (tisagenlecleucel and axicabtagene ciloleucel) have also demonstrated significant clinical responses in hematological cancers (Miller and Sadelain, 2015[46]). However, although modern immunotherapies have arguably shown the most profound effect in cancer treatment, only about 20 % of patients has achieved true cancer cure with these therapies (Schadendorf et al., 2017[53]) (Figure 2(Fig. 2)). 

## Results of the Current Paradigm

“Those diseases which medicines do not cure, iron cures; those which iron cannot cure, fire cures; and those which fire cannot cure, are to be reckoned wholly incurable” (Hippocrates, Aphorisms, Section 7 A87) (Scholtz, 1941[58]). 

**Figure 3:**
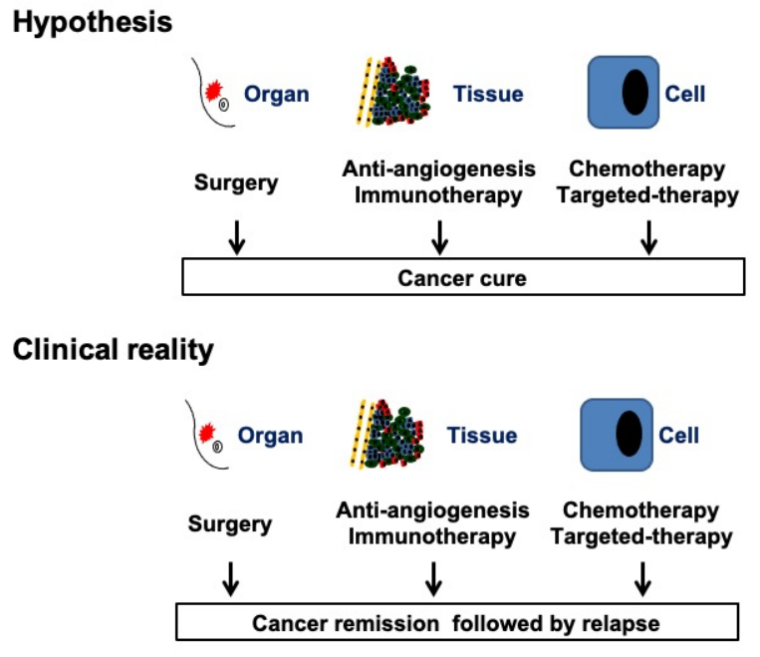
Therapeutic approaches currently used in cancer treatment. According to the “tumor-centric” hypothesis, a cancer cure is only achieved after the complete eradication of the tumor burden. At present, treatments include local (surgery and radiotherapy) and systemic modalities (chemotherapy, hormonal therapies, targeted therapies, angiogenic therapies and immunotherapies). This strategy is not leading to cancer cure (hypothesis) but to cancer remission (clinical situation).

The goal of all medical activity is to prevent illness and cure patients, that is, to preserve health in fit people, and to restore a state of complete physical, mental and social wellbeing in sick people. Our current therapeutic strategy is based on the fact that a cancer cure can only be achieved through the complete eradication of the tumor burden. Based on this and following the concept of the “poisoned arrow” described by Ehrlich, finding the “Achilles heel” of tumors that may be exploited as a specific target whilst sparing healthy tissues (“magic bullet” strategy) becomes very important. At present, these vulnerabilities include specific targets in cancer cells and tumor tissues and are being targeted by local treatments (surgery and radiotherapy) and systemic treatments (chemotherapy, hormonal therapies, targeted therapies, angiogenic therapies and immunotherapies). Indeed, the past decade has witnessed an explosion of combinations of these therapies. This strategy has led to a substantial improvement in survival rates for cancer patients and recently, the cancer mortality rate has dropped in an unprecedented way (Kort et al., 2009[35]). Despite this progress, more than 9 million people still die from cancer every year (IARC, 2019[30]). In addition, cancer survivors suffer chronic morbidities that impair their quality of life (Hudson et al., 2013[29]; Jaffee et al., 2017[31]). We must then admit that, in most patients, the “magic bullet” strategy is not leading to a cancer cure, but to a cancer remission, that is, “the temporary absence of manifestations of a particular disease” (Del Paggio et al., 2017[14]; Sullivan et al., 2017[62]; Thesaurus, 2019[63]). Certainly, current treatments prolong the life of cancer patients and improve their quality of life. We cannot vilify these impacts on every patient life. Any additional time gained with the current treatments can mean a lot to a patient with the prospect of dying. However, inducing a remission is not the same as curing cancer. After months or years of remission, cancer will inevitably recur (Figure 3(Fig. 3)). We can continue looking for other vulnerabilities in tumors, but the problem will persist. 

Therefore, the factual question remains unanswered: how can we truly cure cancer? We can only find the answer to that question if we accept that our current cancer treatment paradigm is obsolete (Galmarini, 2020[23]; Galmarini and Lucius, 2020[24]). The evaluation of the present paradigm shows many triumphs in basic and clinical research, but unfortunately, continues to fail in our goal of restoring a state of complete physical, mental and social wellbeing in most cancer patients. To cure the approximately 18 million people with cancer worldwide, we must shift from this paradigm (IARC, 2019[30]). It is time to pause and think about the key challenges and future directions in clinical oncology.

## Conclusions

There is no doubt that throughout history, the current paradigm has significantly improved cancer care. Today, patients with cancer live longer and with better quality of life than in the past. But the goal of curing all cancers has not been accomplished yet. Currently, cancer therapy is still based on the thousand-year-old paradigm that states that for a cure, complete eradication of cancer cells must be achieved. According to the Hippocratic view, any treatment modality should consider the patient as a unique physical, mental and social entity (Sakula, 1984[52]). We need to regain his wisdom. We need to recover the genius, scientific audacity and true innovation of those who, more than 100 years ago, devised and laid the foundations of modern treatments. It is necessary to integrate genetic, biological, clinical, psychological and social information into a new coherent framework or paradigm to transform it into knowledge and wisdom applied to the clinic that would lead to restoring cancer patients to their fullest physical, emotional, and social capacities. The new paradigm for cancer treatment should be based on this holistic view (Galmarini, 2020[23]). As the old “Masters of Medicine” said, we must treat patients, not illnesses. The real individualization of cancer treatment consists in treating each individual patient following the good general practices of oncology and taking into consideration his/her own particular needs.

## Conflict of interest

Carlos Galmarini is the founder of Topazium Artificial Intelligence.

## Figures and Tables

**Table 1:**
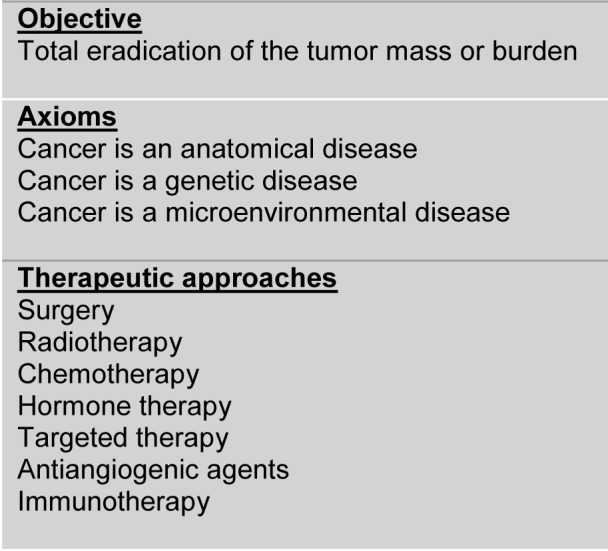
The current cancer treatment paradigm: “For extreme diseases, extreme methods of cure”
